# Social Determinants of Health in Maryland Hip Arthroscopy Patients

**DOI:** 10.7759/cureus.52576

**Published:** 2024-01-19

**Authors:** Parimal Rana, Jane C Brennan, Andrea H Johnson, Justin J Turcotte, Benjamin M Petre

**Affiliations:** 1 Orthopedic Research, Anne Arundel Medical Center, Annapolis, USA; 2 Orthopedics, Anne Arundel Medical Center, Annapolis, USA; 3 Orthopedic Surgery, Anne Arundel Medical Center, Annapolis, USA

**Keywords:** patient-reported outcome measures, anxiety, depression, social determinants of health (sdoh), femoroacetabular impingement syndrome (fais), hip arthroscopy

## Abstract

Background

Prior studies have demonstrated racial and socioeconomic disparities in patient-reported outcome measure (PROM) completion rates, and improvement exists across multiple orthopedic conditions. The purpose of this study was to assess whether these disparities are present in patients undergoing hip arthroscopy (HA) procedures.

Methods

A retrospective study of 306 patients undergoing HA from 2021 to 2023 was performed. Social determinants of health (SDOH) were compared between HA patients and the general Maryland population. Patients were then classified by whether they completed baseline and six-month PROMs (Patient-Reported Outcomes Measurement Information System Physical Function (PROMIS-PF) instrument). Patients who completed PROMIS-PF were classified by whether or not they achieved minimal clinically important difference (MCID) at six months. Demographics and SDOH were compared using univariate analyses between patients who did and did not complete PROMs and between those who did and did not achieve MCID. SDOH were evaluated at the zip-code level using regional health information exchange measures.

Results

Compared to the Maryland population, HA patients resided in areas of lower social vulnerability. Preoperative and six-month PROMs were completed by 102 (33%) patients. No significant differences in demographics or any SDOH were found between patients who did and did not complete PROMs. Six-month MCID was achieved in 75 of 102 (74%) patients with complete PROMs; no significant differences in demographics or SDOH were observed between patients who did and did not achieve MCID.

Conclusions

For patients undergoing HA, disparities in patient-reported outcome completion rates and postoperative functional improvement do not appear to be present across demographics and SDOH, indicating equitable care is being delivered.

## Introduction

Patient-reported outcome measures (PROMs) have the potential to involve both patients and clinicians in enhancing care, research, postoperative outcomes, and healthcare policies. However, their ability to positively affect population health might be restricted due to obstacles that hinder access and effectiveness of these types of measurement systems. Social determinants of health (SDOH) are nonmedical factors, including nonmedical elements that significantly influence PROM completion rates and outcomes. These factors encompass the environments where patients reside, their age, race, education, and employment status, reflecting various influences on living conditions [1]. Previous research has shown racial and socioeconomic disparities in PROM completion rates and outcomes across various orthopedic conditions [2-4]. Studies have shown that patients of increased age, minority race, and non-English speaking status are less likely to complete the PROM questionnaire [3]. This disparity highlights the presence of an underserved population, prompting a study into whether modifications are necessary to enhance patient outcomes.

While prior studies have established the relationship between SDOH and PROM completion rates in other orthopedic populations, a paucity of evidence evaluating this relationship in patients undergoing hip arthroscopy (HA) exists. The purpose of this study is to explore the relationship between socio-demographic factors and postoperative recovery in HA patients by examining the association between patients' social vulnerability, PROM completion rates, and the attainment of clinically significant improvement postoperatively. We hypothesize that HA patients residing in areas of increased social vulnerability will be less likely to complete PROMs and will experience less postoperative improvement in function, in alignment with other patient populations.

## Materials and methods

Study population

This study was deemed exempt by the institutional review board. A retrospective study of 306 patients undergoing HA at a single institution with a single fellowship-trained HA surgeon from 2021 to 2023 was performed. All patients who underwent HA during the study time period and resided in the state of Maryland were included in the study. Any patient residing outside of the state of Maryland was excluded. No other exclusion criteria were used.

Independent variables

Demographics collected were age, sex, body mass index (BMI), and race. Race was classified as white and non-white, based on the low number of non-white patients that were not black or African American (n = 5). The comorbidity burden was evaluated using the American Society of Anesthesiologists (ASA) score. SDOH were evaluated based on the patients’ zip code of residence. The Chesapeake Regional Information System for Our Patients (CRISP) health information exchange was used to quantify various social risk factors in each zip code. The social determinants evaluated included rates of no high school (HS) diploma, unemployment, poverty, no vehicle within the household, crowding, limited English speaking, minority population, children, residents age 65+, and per capita income.

Outcome measures

The primary outcomes of interest were whether patients completed the Patient-Reported Outcomes Measurement Information System Physical Function (PROMIS-PF) instrument preoperatively and at six months postoperatively, and rates of minimal clinically important difference (MCID) achievement. The PROMIS-PF questions ask patients to rate the extent to which their health limits the ability to complete various activities such as running, lifting heavy objects, walking more than a mile, climbing one flight of stairs, lifting or carrying groceries, chores such as vacuuming or yard work, and dressing. Patients' responses to the 10 questions are converted to t-scores, with higher scores indicating greater levels of functional ability. Across the United States population, PROMIS-PF scores are normalized to a mean of 50 with a standard deviation of 10 [5].

MCID was defined as a three-point improvement in PROMIS-PF t-scores from the preoperative to postoperative period, based on the findings of Bodendorfer et al. [6]. PROMIS-PF t-scores at six months postoperatively were evaluated as a secondary outcome. All patients were asked to complete paper versions of the PROMIS-PF instrument during clinic visits, but completion was not required.

Statistical analysis

The various SDOH captured by CRISP were compared between HA patients and the general Maryland population. Demographics and SDOH were then compared using univariate analyses (two-sided independent samples t-tests for continuous and chi-square tests for categorical measures) between patients who did and did not complete PROMs and between those who did and did not achieve MCID. A correlation matrix was created to determine the relationships that exist between the six-month PROMIS-PF score and SDOH. Only correlations that were significant (p < 0.05) were displayed. All statistical analyses were performed using RStudio version 4.2.2 (RStudio, PBC, Boston, MA). Statistical significance was assessed at p < 0.05.

## Results

**Figure 1:**
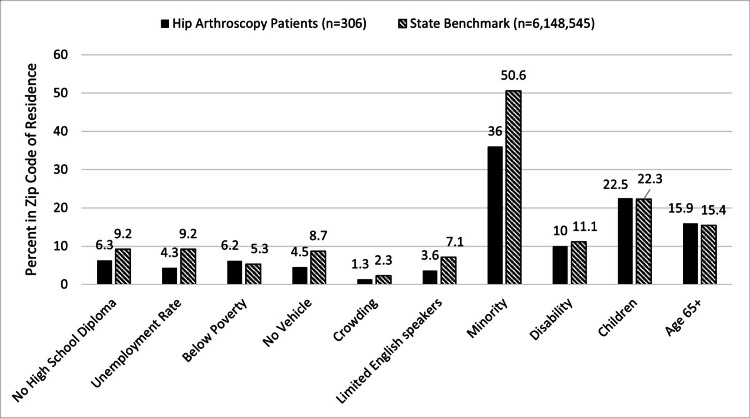
Social determinants of health in hip arthroscopy patients vs. state benchmarks

Compared to the Maryland population, HA patients resided in areas of lower social vulnerability. HA patients resided in zip codes with lower rates of no HS diploma, unemployment, no vehicle, crowding, limited English speaking, and minority population (Figure 1).

Overall, of the 306 HA patients, preoperative and six-month PROMs were completed by 102 (33%) patients. There were no significant differences in age, sex, BMI, race, or ASA scores between those who completed their PROMs and those who did not (Table 1).

**Table 1:** Demographics by PROMIS-PF completion status All data are presented as mean ± SD or n (%). PROMIS-PF, Patient-Reported Outcomes Measurement Information System Physical Function instrument; BMI, body mass index; ASA, American Society of Anesthesiologists score.

Demographics	Did not complete PROMIS-PF (n = 204)	Completed PROMIS-PF (n = 102)	P-value
Age, years	40.1 ± 15.4	41.0 ± 16.4	0.623
Sex			0.862
Female	140 (68.6)	69 (67.6)	
Male	64 (31.4)	33 (32.4)	
BMI, kg/m^2^	27.7 ± 5.6	27.8 ± 5.6	0.909
Non-White	40 (21.1)	23 (23.5)	0.638
ASA	1.9 ± 0.7	1.8 ± 0.6	0.273

Additionally, there were no significant differences in any of the 10 SDOHs evaluated between those who completed their PROMs and those who did not (Table 2).

**Table 2:** Social determinants of health by PROMIS-PF completion status All data are presented as mean ± SD. PROMIS-PF, Patient-Reported Outcomes Measurement Information System Physical Function instrument; HS, high school; USD, United States dollars.

Social determinants of health	Did not complete PROMIS-PF (n = 204)	Completed PROMIS-PF (n = 102)	P-value
No HS diploma (%)	6.4 ± 3.6	6.1 ± 3.4	0.438
Unemployment rate (%)	4.3 ± 1.6	4.4 ± 1.5	0.459
Below poverty rate (%)	6.2 ± 4.1	6.2 ± 4.4	0.973
Weighted per capita income (USD)	52,715 ± 12,039	51,108 ± 11,110	0.261
No vehicle (%)	4.4 ± 4.3	4.6 ± 6.3	0.696
Crowding (%)	1.3 ± 1.3	1.4 ± 1.1	0.630
Limited English (%)	3.6 ± 3.1	3.5 ± 3.0	0.704
Minority (%)	35.0 ± 24.1	37.9 ± 22.9	0.800
Children (%)	22.3 ± 4.0	22.8 ± 4.1	0.251
Age 65+ (%)	16.2 ± 4.6	15.4 ± 5.0	0.144

Of those who completed their PROMs, six-month MCID was achieved by 75 (74%) patients. There were no significant differences in demographics between those who achieved MCID and those who did not. However, patients achieving MCID had lower ASA scores at the time of surgery (1.7 vs. 2.1, p = 0.005) than those who did not (Table 3).

**Table 3:** Demographics by six-month PROMIS-PF MCID achievement All data are presented as mean ± SD or n (%); p-value < 0.05 in bold. PROMIS-PF, Patient-Reported Outcomes Measurement Information System Physical Function instrument; MCID, minimum clinically important difference; BMI, body mass index; ASA, American Society of Anesthesiologists score.

Demographics	No MCID (n = 27)	MCID (n = 75)	P-value
Age, years	44.0 ± 13.0	39.7 ± 17.5	0.187
Sex			1
Female	18 (66.7)	50 (67.6)	
Male	9 (33.3)	24 (32.4)	
BMI, kg/m^2^	29.2 ± 6.0	27.2 ± 5.5	0.126
Non-White	7 (25.9)	15 (20.0)	0.866
ASA	2.1 ± 0.6	1.7 ± 0.6	0.005

Additionally, there were no significant differences in SDOH between those who achieved MCID and those who did not (Table 4).

**Table 4:** Social determinants of health by six-month PROMIS-PF MCID achievement All data are presented as mean ± SD. PROMIS-PF, Patient-Reported Outcomes Measurement Information System Physical Function instrument; MCID, minimum clinically important difference; HS, high school; USD, United States dollars.

Social determinants of health	No MCID (n = 27)	MCID (n = 75)	P-value
No HS diploma (%)	5.5 ± 2.5	6.3 ± 3.7	0.213
Unemployment rate (%)	4.6 ± 1.3	4.4 ± 1.5	0.490
Below poverty rate (%)	5.7 ± 3.2	6.3 ± 4.8	0.453
Weighted per capita income (USD)	52,392.5 ± 11,340.8	50,639.3 ± 11,064.9	0.492
No vehicle (%)	3.6 ± 2.7	5.0 ± 7.2	0.161
Crowding (%)	1.2 ± 0.8	1.5 ± 1.2	0.268
Limited English (%)	3.0 ± 1.8	3.6 ± 3.3	0.221
Minority (%)	36.5 ± 22.3	38.4 ± 23.3	0.708
Children (%)	23.1 ± 4.2	22.7 ± 4.0	0.736
Age 65+ (%)	15.5 ± 5.6	15.3 ± 4.8	0.859

**Figure 2:**
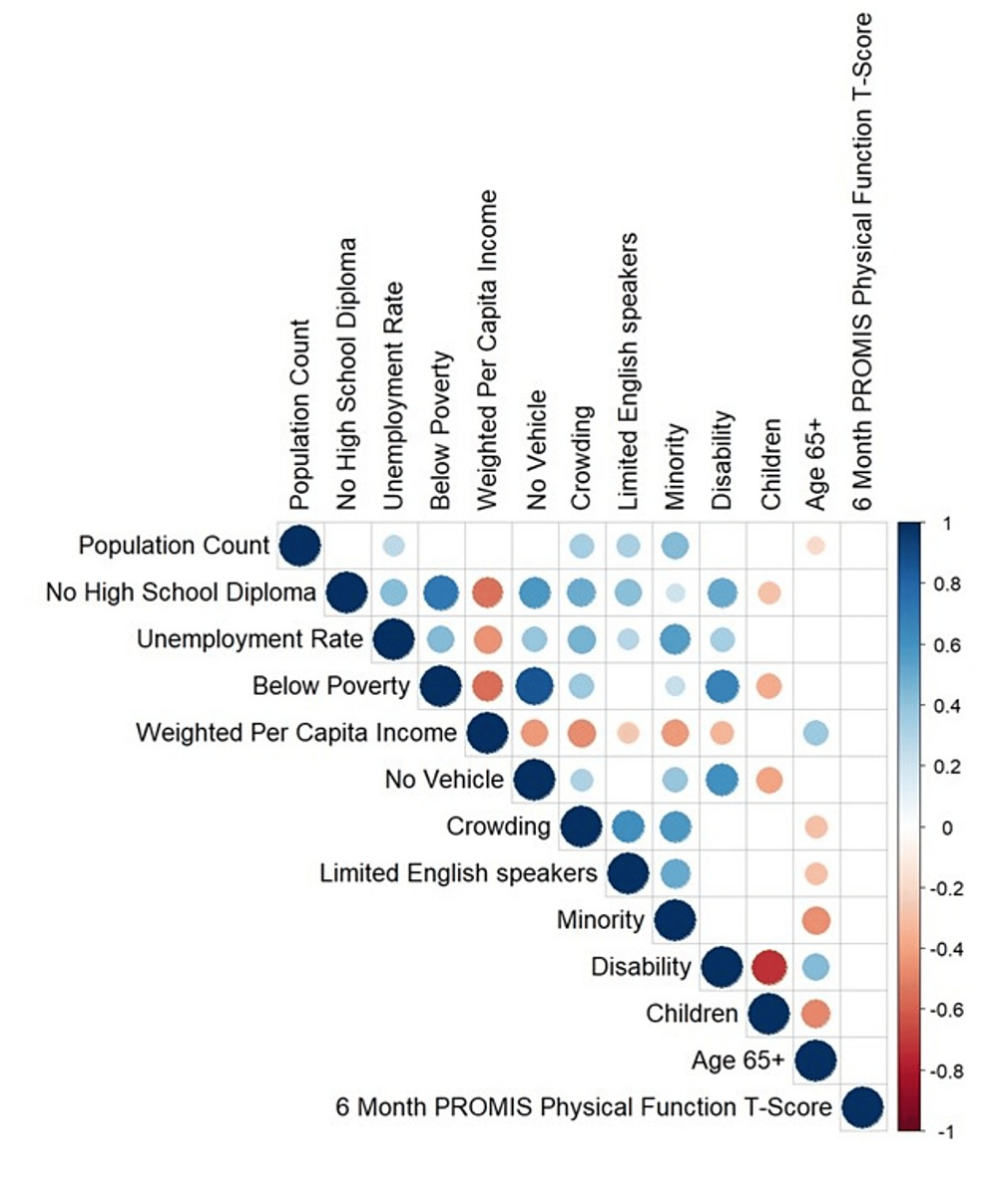
Correlation matrix – social determinants of health vs. six-month PROMIS-PF score Positive correlations are displayed in blue and negative correlations in red. Color intensity and the size of the circle are proportional to the correlation coefficients. On the right side of the correlogram, the legend color shows the correlation coefficients and the corresponding colors. PROMIS-PF, Patient-Reported Outcomes Measurement Information System Physical Function instrument.

Finally, the correlation matrix showed that none of the SDOH measures were significantly correlated with the six-month PROMIS-PF score, although significant relationships between the various social determinants were observed (Figure 2).

## Discussion

HA is a minimally invasive procedure that can reduce pain and increase mobility in various conditions, including femoroacetabular impingement syndrome (FAIS) and labral tears [7]. Despite the increased utilization of this procedure, there is limited data exploring which patients are more likely to benefit from HA procedures [8,9]. Many institutions have adopted PROMs to evaluate the efficacy of HA from the patient's perspective; nevertheless, various barriers may confound or obscure these outcomes. The results from this study suggest that disparities in various SDOH exist between HA patients compared to the general population of Maryland, yet these social determinants did not affect their participation in completing PROMs or achieving MCID post-surgery. Although further study of overall access to care across the region is needed, individuals living in more socially vulnerable regions within Maryland may have a reduced likelihood of undergoing HA or face limited access to this procedure, consequently leading to their exclusion from these data. Nonetheless, the lack of significant associations between SDOH and PROMs completion rates or achievement of clinically significant improvement postoperatively suggests that for patients undergoing HA, equitable care is being delivered across our geographic region.

To comprehensively address disparities in PROM completion, it is essential to examine the broader context of access to orthopedic care. Insurance type, scheduling limitations, and patients' geographic locations can significantly influence the timeliness and accessibility of orthopedic care [10-12]. Although significant changes to Medicaid and efforts to lower the number of uninsured Americans have been implemented in the last decade, studies have shown that access to orthopedic care for Medicaid patients is still significantly limited when compared to privately insured [10,13,14]. Providing specialized care in rural areas is also challenging and creates a burden on the patient to seek access to specialists in their area [15,16]. An estimated 60 million patients living in rural US have limited access to orthopedic care, and that number continues to grow [11]. Shortages of orthopedic providers have only worsened waiting time and access in these patient populations [11,12]. Data have shown that the population’s need for orthopedic services is outpacing the number of providers, increasing the gap between supply and demand each year [17]. Understanding these elements is crucial for understanding and potentially mitigating disparities in PROM completion rates in orthopedics.

Studies exploring PROM completion rates have found that women, patients of white race, and those in the high-income quartile tend to have higher completion rates [18]. Long et al. found that certain patient-level barriers hindered completion rates, including patients' technological literacy, vision, and cognitive functioning [19]. Patients also identified that surveys requiring less time, such as weekly versus daily, were less burdensome and would result in a higher likelihood of completion [20]. A systematic review analyzed paper assessments versus electronic PROMs, finding that electronic PROMs had faster completion, decreased costs, and improved data quality even though technology literacy and access-related barriers existed [21]. Unfortunately, none of the aforementioned studies evaluated PROM completion rates in patients undergoing HA, specifically. Our finding that social determinants were similar among PROM completers and non-completers in this population may be due to multiple factors. First, it is possible that the generally younger age and relatively higher socioeconomic status (SES) of the HA patient population in comparison to the general Maryland population mitigated some of the barriers to PROM completion identified in prior studies. Alternatively, it may be that our collection of PROMs using paper instruments at clinic visits reduced the technological literacy and access hurdles disproportionately faced by those residing in areas of increased social vulnerability. Despite the similar SDOH profiles of patients who did and did not complete PROMs, our relatively low completion rate of 33% at six months postoperatively suggests barriers to completion remain to be addressed.

Limited literature has explored various facets of SDOH and their potential influence on postoperative outcomes in HA patients, demonstrating the importance of completing these assessments. Saks et al. collected PROMs from over 600 patients preoperatively and at their two-year follow-up after HA for FAIS and/or labral tears and concluded that SES had a limited impact on HA outcomes [22]. There were no statistically significant differences observed among any of the groups, stratified by their social deprivation index, regarding preoperative or postoperative outcome scores. All groups exhibited considerable improvements in their PROMs from preoperative to postoperative assessments. No statistically significant differences were identified between any of the groups concerning achieving MCID or patient acceptable symptom state (PASS) rates [22]. Maempel et al. similarly analyzed 89 hip arthroscopies and utilized the Scottish Index of Multiple Deprivation as a measure of social deprivation. Their findings indicated that social deprivation did not serve as a predictive factor for one-year postoperative scores on the International Hip Outcome Tool-12 and EuroQol 5 Dimensions [23]. These studies collectively align with our findings, suggesting that SES may not necessarily impact HA outcomes. Conversely, socioeconomically disadvantaged patients undergoing arthroscopic rotator cuff repairs were more likely to report lower PROM and less likely to reach MCID in previous studies, providing more utility in this patient population [4,24]. While further study is needed to confirm these findings in HA patients, they suggest interventions targeting SDOH may be better targeted to other orthopedic conditions.

Psychosocial factors such as depression and anxiety have shown associations with worse baseline function and poorer outcomes post-surgery [25]. Patients with symptoms of depression have consistently reported lower initial functional scores, higher pain levels, and reduced satisfaction following HA [26]. Martin et al. found that patients exhibiting symptoms of depression not only presented lower initial scores in self-reported function, pain levels, and satisfaction but also maintained these lower scores in the two-year post-surgery follow-up [26]. Specifically, individuals with moderate or severe depression before HA showcased inferior improvements in various functional scores compared to those with minimal or mild depression, and these authors have emphasized the need for counseling referrals as part of the clinical care model [27]. Another study found that undergoing HA for FAIS is still beneficial for this patient population, as it is associated with reduced healthcare resource utilization for treating depression and anxiety postoperatively, indicating a potential alleviation of the psychological healthcare burden after surgery [28]. On the other hand, a systematic review highlighted that preoperative mental health conditions, particularly depression, could lead to suboptimal outcomes in the medium term following arthroscopic hip surgery for FAIS [29].

This relationship becomes crucial when examining the connection between mental health problems and lower SES, as individuals with lower SES have long been acknowledged to experience a disproportionate burden of health concerns, particularly psychological issues [30,31]. Multiple studies have consistently highlighted a higher prevalence of depression among those with lower levels of SES [31,32]. Similarly, studies have indicated an increased prevalence of anxiety within lower SES populations, reporting a 1.5-3 times higher incidence of both anxiety and depression within lower-income communities [30]. In our study, HA patients tended to be less socially vulnerable, leading us to infer that mental health issues might have been less likely to disrupt patient-reported measures in this cohort if compared to the general Maryland population. Nevertheless, these findings emphasize the importance of addressing psychosocial aspects pre- and post-HA, suggesting the potential for tailored interventions to enhance patient outcomes and overall well-being in this patient population.

There are multiple limitations to this study that must be considered. The study’s single-institution focus, limited population size, and potential loss to follow-up should be acknowledged. The study's retrospective nature has inherent limitations and recall bias may exist in the PROMs. It is crucial to acknowledge that these data may not encompass hidden disparities among patients who might lack access to HA procedures due to insurance limitations or geographical barriers, potentially introducing a selection bias and leading to the exclusion of certain groups from this study. These constraints might restrict the generalizability of the findings and underscore the need for further multi-center investigations with larger sample sizes to comprehensively understand the intricate relationship between SDOH and postoperative outcomes in HA patients.

## Conclusions

In individuals undergoing HA, there are no evident discrepancies in completion rates of PROMs or the improvement of functional status after surgery across different demographics and SDOH. Further studies are warranted to examine larger patient cohorts and thoroughly investigate the obstacles impeding the completion of PROMs.
